# Pet and Stray Dogs as Reservoirs of Antimicrobial-Resistant *Escherichia coli*

**DOI:** 10.1155/2021/6664557

**Published:** 2021-01-25

**Authors:** Laura Marchetti, Daniel Buldain, Lihuel Gortari Castillo, Andrea Buchamer, Manuel Chirino‐Trejo, Nora Mestorino

**Affiliations:** ^1^Laboratorio de Estudios Farmacológicos y Toxicológicos (LEFyT), Facultad de Ciencias Veterinarias, Universidad Nacional de La Plata, 60 y 118, 1900 La Plata, Argentina; ^2^Consejo Nacional de Investigaciones Científicas y Técnicas CONICET, Buenos Aires, Argentina; ^3^Department of Veterinary Microbiology, Western College of Veterinary Medicine, University of Saskatchewan, 52 Campus Dr, Saskatoon SK S7N 5B4, Canada

## Abstract

The close contact between dogs and humans creates the best bridge for interspecies transmission of antimicrobial-resistant bacteria. The surveillance of its resistance including the detection of extended-spectrum beta-lactamases (ESBLs) in *Escherichia coli* as indicator bacteria is an important tool to control the use of antimicrobials. The aim of this research was to evaluate the *E. coli* resistance in strains by phenotypic methods, isolated from pet and stray dogs of La Plata city, Argentina. Faecal samples were collected using rectal swabs from 50 dogs with owners (home dogs = HD) and 50 homeless dogs (stray dogs = SD). They were cultured in 3 MacConkey agar plates, with and without antibiotics (ciprofloxacin and cefotaxime). 197 strains were isolated, of which only 95 strains were biochemically identified as *E. coli*, 46 strains were from HD, and 49 were from SD. Antimicrobial susceptibility was evaluated by the Kirby–Bauer disk diffusion method. The most prevalent resistance was for tetracycline, streptomycin, and ampicillin. In both groups, the level of resistance to 3rd generation cephalosporins was high, and there were multiresistant strains. There was a higher level of antimicrobial resistance in strains from SD compared to HD. There were 8% of strains suspected of being ESBLs among samples of HD and 36% of SD. One (2%) of the strains isolated from HD and 11 (22%) from SD were phenotypically confirmed as ESBL. Pets and stray dogs are a potential source of *E. coli* antibiotic resistance in Argentina; therefore, its surveillance must be guaranteed.

## 1. Introduction

The role of pets as one of the most important disseminators of antimicrobial-resistant bacteria has been underestimated for a long time because the general focus has been aimed at food-producing animals as the principal source of resistant bacteria. However, the close contact between pets and humans creates the best bridge for interspecies transmission of multidrug-resistant (MDR) bacteria [1].

Dogs and cats which live in the same house with their owners make contact with the same surfaces and objects, and these habits increase the chances of antimicrobial resistance dissemination. Furthermore, veterinary practice evolution and the increasing sense of social responsibility for the welfare and health of pets have enhanced their life expectancy, which has increased the number of geriatric patients that frequently need antimicrobial therapy, since they often suffer from chronic diseases or immunocompromising conditions [2].

Antimicrobials are frequently used for therapeutic and prophylactic purposes, not only in dogs that live in homes but also in stray dogs. In Argentina, people without professional knowledge frequently administrate antimicrobials to stray dogs because of the unrestricted antibiotic sale and the increasing tendency to protect animals [3]. The inadequate use and abusive prescription of antibiotics, the application of subtherapeutic doses, the irregularity in the administration of antimicrobials, and the change in the social role of dogs among the community nowadays are important risk factors for antimicrobial resistance selection and the transference of bacteria with gene resistance determinants. Several studies have proved that antimicrobial-resistant bacteria could be transferred from dogs to humans and from humans to dogs [4–6].

In recent years, there has been an important increment in the number of companion animals in Argentina. According to a recent study of the Department of Health and Animal Protection of the Government of Buenos Aires city, there are between 800000 and one million dogs and cats, with and without owners. This means that there is one pet for every 3 humans [7, 8].

Several resistance microorganisms have been isolated from healthy and sick pets, such as methicillin-resistant *Staphylococcus aureus* (MRSA), methicillin resistance *Staphylococcus pseudintermedius* (MRSP), multidrug-resistance Gram-negative bacteria, and extended-spectrum beta-lactamase (ESBL)/AmpC-producing *Enterobacteriaceae* [9, 10]. The great increase in strains carrying ESBLs in humans, animals, and their surrounding environments is a huge problem worldwide. Companion animals would have a possible role as reservoirs for ESBLs [11].

ESBL genes in *Escherichia coli* are mainly encoded in plasmids; nevertheless, there are studies that confirmed chromosomal integration [12–14]. These enzymes have hydrolytic activity against penicillins, third-generation cephalosporins (ceftazidime or cefotaxime), and aztreonam, but not the cephamycins (cefoxitin) or carbapenems, and are inhibited by beta-lactamase inhibitors as clavulanic acid [15]. Beta-lactams are possibly the antimicrobials most widely used not only in human medicine but also in animals due to the safety, antimicrobial spectrum, availability, and pharmacokinetic and pharmacodynamic properties [16]. First-generation cephalosporins and amoxicillin with clavulanic acid and penicillin with an aminoglycoside are prescribed routinely in daily veterinary clinic practice. Third-generation cephalosporins represent one of a few therapeutic options to treat bacterial infections of difficult resolution; therefore, they are critically important drugs.

*Enterobacteriaceae* are the main producers of ESBL, particularly *Klebsiella pneumoniae* and *E. coli* [17]. Among the intestinal microbiome, *E. coli* has a role as an indicator of resistance because it is one of the most widespread groups of intestinal bacteria. The surveillance of its resistance mechanisms is an important tool in the control of nonprudent use of antimicrobials. It allows us to know which antimicrobials are selected for antimicrobial resistance, avoiding public health risks, reducing therapeutic failures and economic losses among producers [5].

Furthermore, ESBL strains often carry antimicrobial resistance genes for other antibiotics, such as fluoroquinolones, aminoglycosides, tetracyclines, sulfamethoxazole-trimethoprim, and chloramphenicol. Consequently, there is a potential risk of cross-resistance mediated by plasmids, and there is a great concern because of the increasing emergence of different phenotypes of resistance such as multidrug resistance (MDR: when at least one agent in three or more families of antibiotics is not sensitive), extreme resistance (XDR) when they are not sensitive to at least one agent in all categories of antibacterial families except one or two of them), and pan-resistance (PDR: when they are not sensitive to any of the agents in all the antimicrobial categories) [18, 19].

The objectives of this research were to detect resistance patterns and *in vitro* ESBLs producing *E. coli* strains by phenotypic methods, isolated from dogs with owners and stray dogs of La Plata city, Buenos Aires, Argentina.

## 2. Materials and Methods

### 2.1. Bacterial Strains

50 faecal samples of dogs living in houses with owners (home dogs = HD) and 50 samples of homeless dogs (stray dogs = SD) were collected from October to December 2016 in La Plata city, Buenos Aires, Argentina. Dogs with owners were clinically healthy, adults, and raised in family homes. They did not receive antimicrobial treatments in the last 3 months. For samples taken in a veterinary clinic, only those animals that come for routine health control or to reinforce vaccines by the health plan were selected. Stray dog faecal samples were collected in different areas of La Plata city following the spiral sampling method [20]. La Plata city is characterized by its particular design because it is a grid, a perfect square with two diagonals that cross the complete city from east to west and from north to south. Consequently, stray dogs were sampled beginning from the peripheral area to the centre of the square. Only nonaggressive stray dogs were chosen for sampling since no sedation method was used. Animals were restrained by two operators, and a third person took the rectal faecal sample using a sterile cotton swab.

The protocol was carried out according to the Guide for the Care and Use of Agricultural Animals in Agricultural Research and Teaching (Federation of Animal Science Societies–FASS-) [21] and was approved by the Experimental Ethics Committee of the Faculty of Veterinary Science, UNLP, Argentina (47.3.15 J).

In the laboratory, each cotton swab was used to inoculate each sample onto 3 agar plates: MacConkey agar (Difco, Becton Dickinson, USA) supplemented with 2 mg/L cefotaxime (plate A), MacConkey agar supplemented with 0.05 mg/L ciprofloxacin (plate B) to select possible resistant strains present in the samples, and the third plate without antimicrobial (plate C). Cefotaxime and ciprofloxacin were obtained from Sigma-Aldrich, MO, USA. After overnight incubation at 37°C, 2-3 colonies with the phenotypic characteristics of *E. coli* were selected, Gram stained, and typified by biochemical tests (Urease production, Catalase test, Motility, Voges Proskauer, Indole production, Carbohydrate fermentation tests, Methyl red, and Citrate utilization). *E. coli* ATCC 25922^®^ (American Type Culture Collection, USA) was used as quality control [22, 23].

### 2.2. Susceptibility Testing

The strains that grew in plates A and/or B were identified biochemically as *E. coli* and tested for antimicrobial susceptibility by the standard Kirby-Bauer disk diffusion method. But when there were no colonies on those plates (A or B) but in C, strains developed in this last plate without antibiotic were isolated to study. Twenty antimicrobials (Becton Dickinson, USA) were selected: amoxicillin/clavulanic acid (20 *μ*g/10 *μ*g), ampicillin (10 *μ*g), ampicillin-sulbactam (10 *μ*g/10 *μ*g), ceftriaxone (30 *μ*g), ceftazidime (30 *μ*g), cefotaxime (30 *μ*g), cefazoline (30 *μ*g), cefpodoxime (10 *μ*g), sulfamethoxazole-trimethoprim (23.75 *μ*g/1.25 *μ*g), chloramphenicol (30 *μ*g), ciprofloxacin (5 *μ*g), nalidixic acid (30 *μ*g), tetracycline (30 *μ*g), gentamicin (10 *μ*g), streptomycin (10 *μ*g), kanamycin (30 *μ*g), amikacin (30 *μ*g), nitrofurantoin (30 *μ*g), aztreonam (30 *μ*g), and imipenem (10 *μ*g), according to the Clinical and Laboratory Standards Institute, CLSI [22, 23].

### 2.3. ESBL Identification

The first ESBL disc screening test was evaluated using antibiotic discs of cefpodoxime, ceftazidime, aztreonam, cefotaxime, and ceftriaxone on Mueller Hinton agar (Britania, Argentine) [18, 19]. Results were interpreted according to CLSI screening cut-off values for the antimicrobials mentioned above. When the diameter around one of the disks mentioned was cefpodoxime ≤17 mm, ceftazidime ≤22 mm, cefotaxime ≤27 mm, ceftriaxone ≤25 mm, aztreonam ≤27 mm, respectively, the isolations were considered suspected of ESBL phenotype [22, 23].

For the ESBL confirmation, the Double-Disc Synergy Test was applied according to CLSI 2013; discs containing ceftazidime and cefpodoxime were put next to a disc with amoxicillin plus clavulanic acid (20 mm centre to centre). The positive result is indicated when the inhibition zones around any of the cephalosporin discs were augmented in the direction of the disc containing clavulanic acid. *E. coli* ATCC 25922^®^ was used for quality control [22, 23].

### 2.4. Statistical Analysis

Chi-square test was used to determine the significance of differences in resistance prevalence between stray dogs and pets. A value of *p* ≤ 0.05 was considered significant.

## 3. Results and Discussion

197 strains were isolated (77 from HD and 120 from SD) from the 100 faecal samples. Only 95 strains biochemically identified as *E. coli* (46 from HD and 49 from SD) were studied; every strain was individual and becomes from one of the 3 plates mentioned above (A, B, or C). From a few samples, we obtained isolations biochemically identified as *E. coli* from both plates A and B at the same time, but we studied them individually. They were chosen as follows: from samples of HD, 3 strains of *E. coli* grew on plate A (with cefotaxime), 19 grew on plate B (with ciprofloxacin), and the rest of the strains (*n* = 24) only developed on agar without antimicrobials; on the other hand, from samples of SD, 19 strains of *E. coli* developed on plate A and 16 on plate B; the rest (*n* = 14) grew in the plates without antimicrobials (plate C).

Those strains that had grown on agar with cefotaxime (plate A) or with ciprofloxacin (plate B) were selected to carry out antibiograms. Strains of *E. coli* that were inhibited in plates A and B but developed in plate C without antimicrobial were the third group studied, including susceptibility test. Therefore, a total of 46 strains of dogs with owner and 49 without owner were selected for the study.

**Figure 1:**
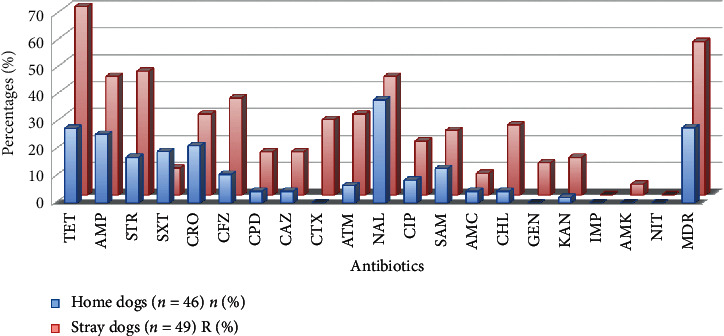
Antimicrobial resistance percentages in home dog and stray dog strains. TET (tetracycline), AMP (ampicillin), STR (streptomycin), STX (sulfamethoxazole-trimethoprim), CRO (ceftriaxone), CFZ (cefazolin), CPD (cefpodoxime), CAZ (ceftazidime), CTX (cefotaxime), ATM (aztreonam), NAL (nalidixic acid), CIP (ciprofloxacin), SAM (ampicillin-sulbactam), AMC (amoxicillin-clavulanic acid), CHL (chloramphenicol), GEN (gentamicin), KAN (kanamycin), IMP (imipenem), AMK (amikacin), NIT (nitofuratoin), MDR (multidrug-resistant).

The most common resistance observed was to tetracycline (70%) in SD-derived strains and to nalidixic acid (38.3%) in HD-derived strains. Resistance to streptomycin (46%) and ampicillin (44%) was the second high in SD strains. For HD, there were 25.5% of resistance to ampicillin and 19.1% to sulfamethoxazole-trimethoprim. In both groups, the level of resistance to 3rd generation cephalosporins was high (ceftriaxone: 30% SD–21.3% HD and cefotaxime: 28% for SD). There was a higher level of antimicrobial resistance in strains from SD compared to HD. No *E. coli* from either group showed resistance to imipenem or nitrofurantoin. There were multiresistant strains in both groups, and their resistance profile includes one, two, three, and more than four antimicrobials (see Figure 1 and Table 1).

There were 8% of strains suspected of being ESBL among samples of HD and 36% of SD. One of the suspected ESBL strains from HD (2%) and 11 (22%) of SD were confirmed.

The most prevalent phenotypes of resistance detected among the *E. coli* isolates recovered from HD were nalidixic acid (NAL = 5 isolates) and nalidixic acid with tetracycline (NAL + TET = two isolates).

The multiresistance occurrence was considerably higher in SD strains, with 32.7% of MDR profiles to more than 4 antimicrobials. The most prevalent phenotypes detected for SD isolates were tetracycline (TET = 8 isolates), and as HD strains, nalidixic acid with tetracycline (NAL + TET = three isolates).

All the strains that showed resistance to 4 antimicrobials or more in both groups (HD and SD strains) belong to isolates that had previously developed in plates with antimicrobial (ciprofloxacin and cefotaxime) (see Tables 2 and 3).

In our study, samples were obtained from faeces of animals with and without owners, but they were not clinical samples because the principal aim of our work was to know the prevalence of antimicrobial resistance in *E. coli* as indicator bacteria in La Plata city, in dogs without recent antimicrobial therapy. Most authors have published results obtained from clinical samples such as urine, urinary tract, intestinal tract, and ears [24–26]. This makes it difficult to compare results since there is an expected difference in the prevalence of resistance between populations of sick and healthy dogs.

Resistance to antimicrobials in the present study appeared to be higher than those previously reported in other studies done with healthy dogs. The highest level of resistance obtained was for tetracycline in SD-derived strains but not in pets (*p* < 0.0001). Ampicillin was the second most prevalent resistance observed (SD = 44%; HD = 25.5%); however, it was not quite significant (*p*=0.8888). Resistance to nalidixic acid was common in both groups of animals (HD = 38.3%; SD = 44%), considered not significant (*p*=0.6798). Stray dog isolates also showed high resistance against streptomycin (46%), but it was low (17%) in pets (*p* < 0.0001). Resistance to ampicillin and tetracycline is the most frequently reported in most studies made with healthy dogs, in different countries [27–29]. The resistance percentages obtained for the antimicrobials mentioned above in pet strains (HD) are similar to those reported in a study of Wedley et al. [27], from healthy dogs living in a semirural community in Cheshire, UK (24% for ampicillin and 19.7% for tetracycline). In another study carried out in dogs visiting veterinarians from the UK, they found a higher level of resistance for both antibiotics; 37.2% of the isolates were resistant to ampicillin and 30% to tetracycline [28]. Nevertheless, these results are still lower than those reported for SD isolates in our work. The results reported by Costa et al. [30], in healthy pets from Portugal isolates, were even lower: 20.5% of resistance for tetracyclines and 7.7% for ampicillin. Interestingly, in some studies done with clinical isolates, in the *E. coli* isolated from urinary infections, septicaemia, and skin and soft tissue infections, the resistance to ampicillin and tetracycline was lower than that of our results [24, 31].

Beta-lactams and tetracyclines are the antimicrobials more used in pets; the different levels of resistance reported in every work correlate with the frequency and magnitude of their use in different countries. Furthermore, our results are disturbing because they demonstrate that in La Plata it seems to be an abuse and/or misuse of broad-spectrum antimicrobials such as tetracyclines, as well as beta-lactams.

The identification of ESBLs producing strains is very important to guide an adequate antimicrobial therapy. There were 8% of strains suspected of being ESBLs among samples of HD and 36% from SD. One of the ESBL strains suspected from HD (2%) and 11 (22%) of SD were confirmed by phenotypic tests, but there was no genetic confirmation, which is a limitation in our study. Again, the results obtained for HD strains are similar to those published by Wedley et al. [27, 28], but the percentage of ESBL in SD strains was considerably higher.

The prevalence of multidrug resistance (resistance to more than 3 antimicrobials) was considerably higher in SD strains, compared to several studies published by other authors [27, 28, 32–34]. In pet strains (HD), there was a similar percentage (28%) of MDR to those reported by Murphy et al. [34], in a study made on healthy dogs from Canada. Again, the findings in SD strains are a big concern. Antimicrobials can be acquired without a professional prescription in Argentina. This is one of the possible reasons that lead to the administration of them by anyone, even without being a veterinarian. Another possible source of antimicrobial resistance transmission is the garbage; stray dogs break the garbage bags and eat them. It is possible that bacteria contained in those bags carry antimicrobial resistance genes. On the other hand, household antibiotic waste contaminates the nonhome environment, selecting antimicrobial resistance in bacteria there, which then infect stray dogs and other humans. In Argentina, data are extremely limited for the amount of antimicrobial-resistant bacteria on pets and in the near-home environment. Furthermore, whilst antimicrobials are monitored in stuff entering the formal market, there is a significant informal market, and antimicrobial levels in the environment are not routinely measured. One potential response to the rising threat of antimicrobial resistance is regulation, and both international organizations—led by the WHO—and individual countries have sought to formulate, for example, new regulatory frameworks to address the use of antimicrobials, as well as the household waste management. The source of the resistance genes in those bacteria of stray dogs is still unknown, and further studies should be done to detect such environmental source(s).

One of the most prevalent phenotypes detected for SD and HD isolates was nalidixic acid with tetracycline (NAL + TET = three isolates). Those strains were NAL^R^ CIP^S^; it means that they were resistant to nalidixic acid and susceptible to ciprofloxacin. This is a frequent finding in daily veterinary clinic laboratory. The mechanisms of resistance to quinolones are target alterations or efflux pump overexpression. The most relevant mechanism is the mutation of genes encoding quinolone targets (DNA gyrase and topoisomerase IV). The mutation on a point of the *gyrA* gene is frequently in *E. coli* clinical strains and has been associated with this phenotype NAL^R^ CIP^S^. Bacteria that have this kind of mutation have shown to develop more frequently higher levels of resistance in the presence of quinolones [35].

## 4. Conclusions

It is very important to know the prevalence of antimicrobial resistance by surveillance studies not only in pets but also in homeless dogs. The unrestricted antibiotic sale, weak management of household waste, and the increasing tendency to protect animals in Argentina are important risk factors for antimicrobial resistance selection and the transference of gene resistance determinants among bacteria from pets, homeless dogs, and humans. The results of the present study indicate that similar studies in other cities of Argentina would be very useful for monitoring antimicrobial resistance at the national level.

## Figures and Tables

**Table 1:** Prevalence of antimicrobial resistance *E. coli* isolated from pets and stray dogs.

Antimicrobial agents	Antimicrobial-resistant *E. coli* isolated from			
	Home dogs (*n* = 46)		Stray dogs (*n* = 49)	
	Number	Percentage	Number	Percentage
Tetracycline^a^	13	27.7	35	70
Ampicillin	12	25.5	22	44
Streptomycin^a^	8	17	23	46
Sulfamethoxazole-Trimethoprim	9	19.1	5	10
Ceftriaxone	10	21.3	15	30
Cefazolin^a^	5	10.6	18	36
Cefpodoxime	2	4.3	8	16
Ceftazidime	2	4.3	8	16
Cefotaxime^a^	0	0	14	28
Aztreonam^a^	4	6.4	15	30
Nalidixic acid	18	38.3	22	44
Ciprofloxacin	4	8.5	10	20
Ampicillin-sulbactam	6	12.8	12	24
Amoxicillin-clavulanic	2	4.3	4	8
Chloramphenicol^a^	2	4.3	13	26
Gentamicin^a^	0	0	6	12
Kanamycin^a^	1	2.1	7	14
Imipenem	0	0	0	0
Amikacin	0	0	2	4
Nitrofurantoin	0	0	0	0
R 1 ATM	8	17.4	9	18.4
R 2 ATMs	5	10.9	5	10.2
R 3 ATMs	2	4.3	5	10.2
R 4 or more ATMs	11	23.9	23	32.7

^a^ *p* ≤ 0.05; R 1 ATM (resistance to only one antimicrobial); R 2 ATMs (resistance to two antimicrobials only); R 3 ATMs (resistance to three antimicrobials only); R 4 or more ATMs (resistance to four or more antimicrobials).

**Table 2:** Phenotypes of resistance detected among the *E. coli* isolates recovered from pets.

Phenotype of resistance	Number of isolates	Percentage of isolates
CRO	1	2.17
STR	1	2.17
ATM	1	2.17
NAL^a^	5	10.8
KAN + NAL	1	2.17
TET + STR	1	2.17
NAL + TET^a^	2	4.3
NAL + CRO	1	2.17
AMC + CFZ + AMP	1	2.17
SXT + CFZ + AMP	1	2.17
SXT + CIP + NAL + TET^a^	1	2.17
STX + NAL + TET + STR^a^	1	2.17
SXT + TET + STR + AMP + CRO^a^	1	2.17
STX + TET + STR + AMP + CRO^a^	1	2.17
NA + TET + STR + AMP + CRO^a^	1	2.17
STX + CFZ + NAL + TET + AMP^a^	1	2.17
SXT + SAM + CIP + NAL + TET + AMP^a^	1	2.17
STX + SAM + CIP + NAL + TET + STR + AMP^a^	1	2.17
STX + SAM + NAL + TET + AMP + CRO + ATM^a^	1	2.17
CTX + CFZ + SAM + CPD + CRO + CIP + NAL + AMP + CRO^b^	1	2.17
CAZ + CTX + AMC + CFZ + SAM + CIP + CRO + AMP + ATM^b^	1	2.17

^a^ *E. coli* developed on the plate with ciprofloxacin; ^b^*E. coli* developed on the plate with cefotaxime. TET, tetracycline; AMP, ampicillin; STR, streptomycin; STX, sulfamethoxazole-trimethoprim; CRO, ceftriaxone; CFZ, cefazolin; CPD, cefpodoxime; CAZ, ceftazidime; CTX, cefotaxime; ATM, aztreonam; NAL, nalidixic acid; CIP, ciprofloxacin; SAM, ampicillin-sulbactam; AMC, amoxicillin-clavulanic acid; GEN, gentamicin; KAN, kanamycin.

**Table 3:** Phenotypes of resistance detected among the *E. coli* isolates recovered from stray dogs.

Phenotype of resistance	Number of isolates	Percentage of isolates
TET ^a(1)^	8	16.3
NA^b^	1	2.04
STX + CZ	1	2.04
NAL + TET^a^	3	6.12
SAM + AMP^a^	1	2.04
CFZ + STR + ATM^a^	2	4.08
TET + STR + CRO	1	2.04
TET + STR + AMP	1	2.04
TET + AMP + CRO^a^	1	2.04
TET + STR + AMP + CRO^a^	1	2.04
CAZ + CTX + NA + TET + STR^b^	1	2.04
CIP + NA + TET + STR + AMP^a^	1	2.04
CIP + NA + TET + STR + AMP + CRO^a^	2	4.08
CAZ + CTX + NA + TET + STR + AMP + CRO^a^	1	2.04
CPD + CIP + NAL + TET + STR + AMP + CRO^a^	1	2.04
SXT + KAN + NAL + TET + GEN + STR + AMP + CRO^a^	1	2.04
CAZ + CTX + CFZ + CRO + KAN + NAL + TET + ATM^b^	1	2.04
CTX + CFZ + SAM + CPD + CRO + KAN + TET + GEN^b^	1	2.04
CAZ + AMC + CFZ + SAM + CPD + TET + STR + AMP + ATM^b^	1	2.04
CAZ + AMK + CFZ + CRO + KAN + NAL + GEN + STR + ATM^b^	1	2.04
CTX + CFZ + SAM + CRO + KAN + TET + GEN + STR + AMP + ATM^b^	1	2.04
CTX + CFZ + SAM + CPD + CRO + NAL + TET + STR + AM + CRO	1	2.04
CTX + CFZ + SAM + CTD + CRO + NA + TET + STR + AMP + CRO^b^	1	2.04
CTX + CFZ + SAM + CRO + KAN + NAL + GEN + STR + AMP + ATM^b^	1	2.04
CTX + SXT + CFZ + CRO + CIP + NAL + TET + STR + AMP + ATM^b^	2	4.08
CTX + CFZ + SAM + CPD + CRO + KAN + TET + GEN + STR + AMP + ATM^a^	1	2.04
CAZ + CTX + AMC + CFZ + SAM + CRO + CIP + NAL + TET + AMP + CRO + ATM^b^	1	2.04
CAZ + CTX + AMC + CFZ + SAM + CRO + CIP + NAL + TET + STR + AMP + CRO + ATM^a.b^	2	4.08
CAZ + CTX + SXT + AMK + CFZ + SAM + CPD + CRO + CIP + NAL + TET + AMP + ATM^a^	1	2.04

^a^ *E. coli* developed on the plate with ciprofloxacin; TET^a(1)^ One *E. coli* developed on the plate with ciprofloxacin; ^b^*E. coli* developed on the plate with cefotaxime. TET, tetracycline; AMP, ampicillin; STR, streptomycin; STX, sulfamethoxazole-trimethoprim; CRO, ceftriaxone; CFZ, cefazolin; CPD, cefpodoxime; CAZ, ceftazidime; CTX, cefotaxime; ATM, aztreonam; NAL, nalidixic acid; CIP, ciprofloxacin; SAM, ampicillin-sulbactam; AMC, amoxicillin-clavulanic acid; CHL, chloramphenicol; GEN, gentamicin; KAN, kanamycin; AMK, amikacin.

## Data Availability

The data used to support the findings of this study are available from Laboratorio de Estudios Farmacológicos y Toxicológicos ‐LEFyT‐, Facultad de Ciencias Veterinarias, Universidad Nacional de La Plata upon request (nmestorino@yahoo.com, noram@fcv.unlp.edu.ar, mlauramarchetti@yahoo.com.ar, and mlmarchetti@fcv.unlp.edu.ar).
